# Case Report: Pulmonary sarcomatoid carcinoma in a female patient from Nepal

**DOI:** 10.12688/f1000research.55187.1

**Published:** 2021-08-02

**Authors:** Anirudra Devkota, Amrit Paudel, Simit Sapkota, Subash Pandit, Aashish Baniya

**Affiliations:** 1 Emergency Department, Patan Hospital, Lalitpur, Nepal; 2 Department of Internal Medicine, Union Memorial Hospital, Baltimore, MD, USA; 3 Department of Oncology, Civil Service Hospital, Kathmandu, Nepal; 4 Department of Neurology, Upendra Devkota Memorial National Institute of Neurological and Allied Sciences (UDM-NINAS), Kathmandu, Nepal

**Keywords:** aggressive, carcinoma, chemotherapy, diagnosis, pulmonary sarcomatoid.

## Abstract

Sarcomatoid carcinoma of the lung is an uncommon subtype of non-small-cell lung cancer (NSCLC). Even in the early stages, pulmonary sarcomatoid carcinoma (PSC) has a dismal prognosis when compared to other kinds of NSCLC with a mean survival of 9–12 months and a five-year survival rate of around 20%. We present the case of a 68-year-old woman with a two-month history of shortness of breath and cough. Initial computed tomography (CT) scan showed features of interstitial lung disease with chronic obstructive airway changes. After 34 months, the patient’s condition worsened with newer complaints of sore throat and hemoptysis. A repeat CT scan showed a ∼49x38x59mm size lesion in the superior segment of the left lower lobe. A core needle biopsy was performed, which revealed tumor cells consisting of irregular tubules and sarcomatoid components. The patient was started on chemotherapy. Unfortunately, she succumbed to her disease. Our case highlights the aggressiveness of PSC.

## Introduction

Sarcomatoid carcinoma (SC) is a rare primary malignant tumor consisting of both carcinomatous and sarcomatous components. It can affect various organs and body parts including skin, bone, urinary tract, breast, pancreas, liver, and glands ( Shen et al., 2013). Pulmonary SC (PSC) accounts for less than 1% of all lung cancers and is known to behave aggressively ( Szkorupa et al., 2015; Travis et al., 2015). It is classified as pleomorphic carcinoma, giant cell carcinoma, spindle cell carcinoma, carcinosarcoma, and pulmonary blastoma. The mean age of diagnosis is 65-75 years and is more common in males, with smoking being the most common risk factor. Here, we report a case of PSC in a 68-year-old woman.

## Case report

**Figure 1.**
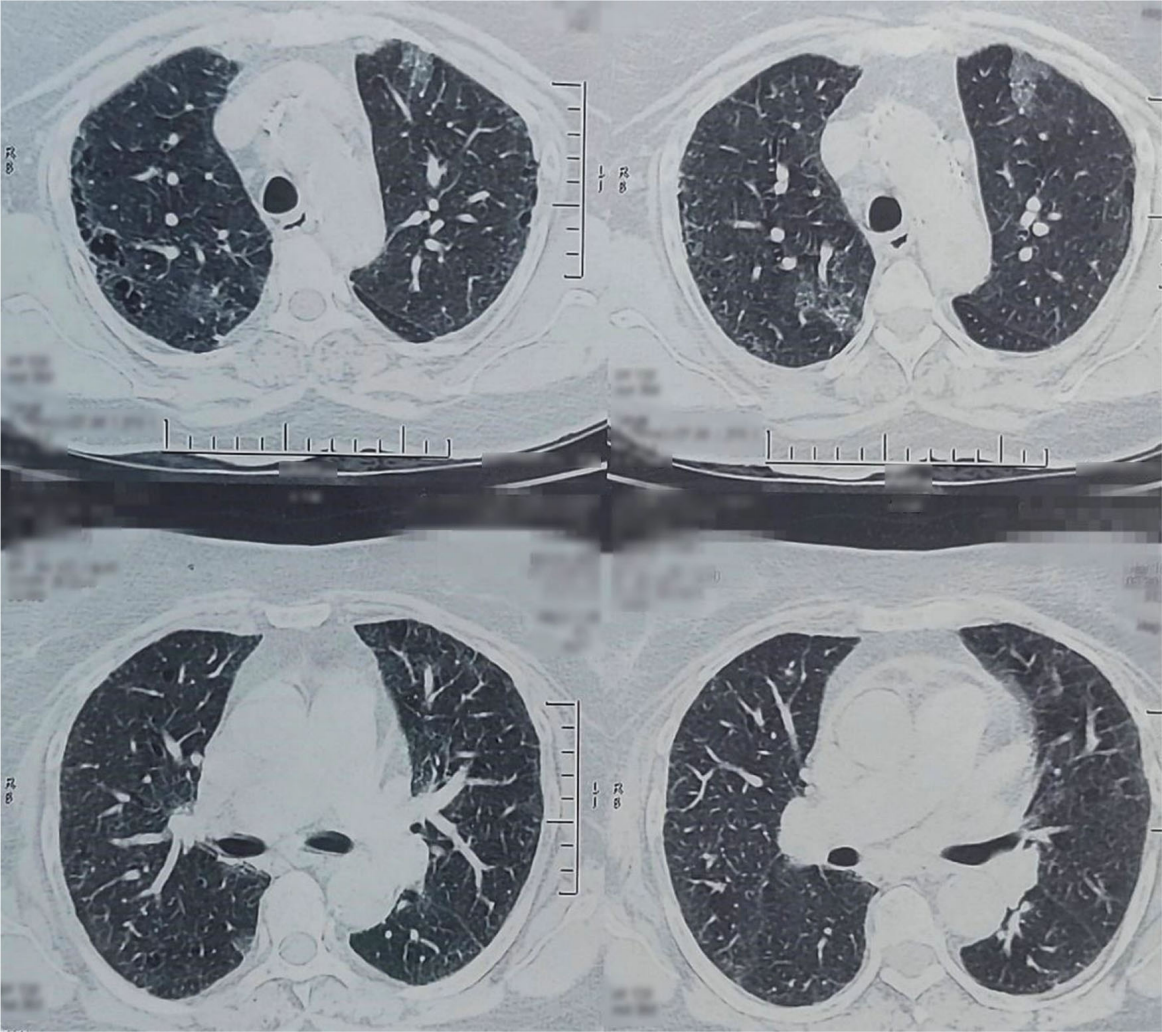
Axial section of computed tomography chest showing features of interstitial lung disease with chronic obstructive airway disease changes.

A 68-year-old Nepali housewife with a 30-cigarette pack per year smoking history presented initially with dyspnea and dry cough of two months. The patient also had a history of long-term exposure to bio-mass fuel. Physical examination revealed clubbing in bilateral fingernails. A chest X-ray was done, which showed bilateral basal region haziness and fibrosis. Computed tomography (CT) scan showed features of interstitial lung disease with chronic obstructive airway disease changes ( Figure 1). A pulmonary function test (PFT) was also performed, which was within normal limits. Hence, the patient was treated for interstitial lung disease (ILD). This led to an improvement in her symptoms and her condition was stable for 34 months.

**Figure 2.**
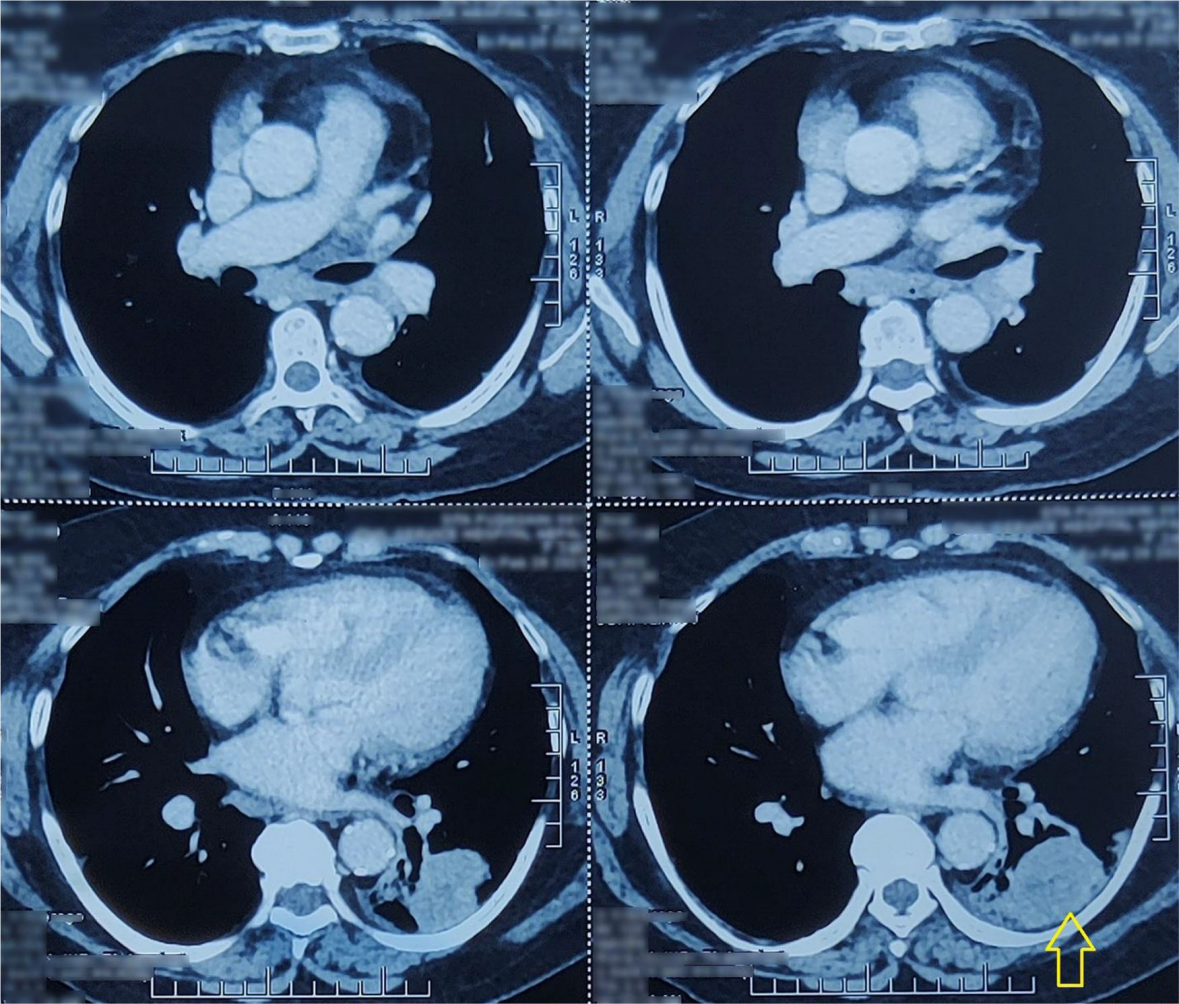
Axial section of computed tomography chest showing heterogeneously enhancing mass in the superior segment of the left lower lobe (arrow) with cavitation and surrounding septal thickening.

**Figure 3.**
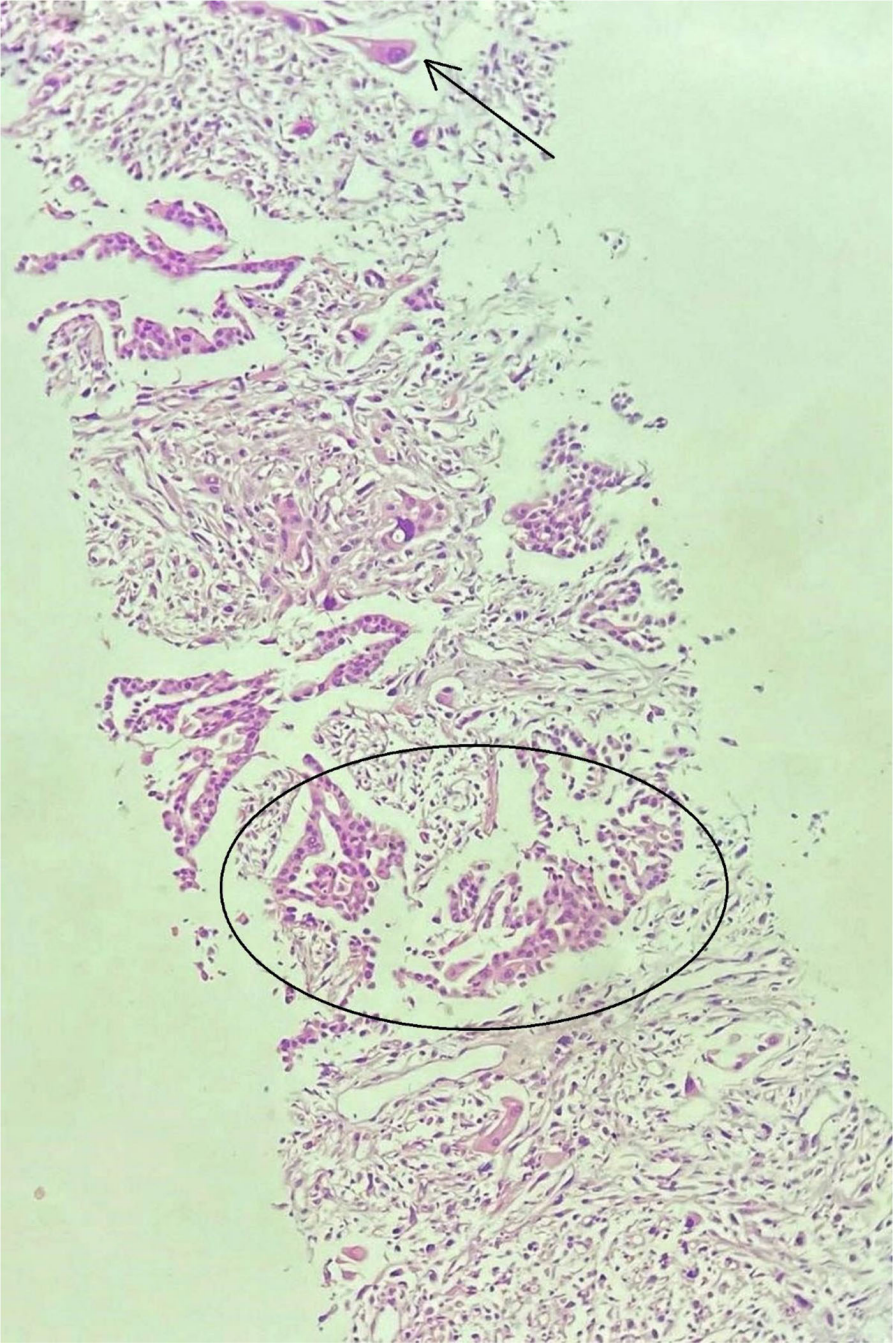
Hematoxylin and eosin-stained slide revealing tumor cells. The circular inlet shows irregular tubules and arrow shows sarcomatoid component (20×).

After 34 months, the patient again started having a dry cough with dyspnea. She also had a few episodes of hemoptysis. She was noted to be hypoxic with an oxygen saturation of 88%. Compared to the previous scan at initial assessment, a contrast-enhanced CT scan of the chest revealed an approximately 49 × 38 × 59 mm size heterogeneously enhancing lesion in the superior segment of the left lower lobe ( Figure 2), extending into posterior basal segment with cavitation and surrounding septal thickening, with consolidation and ground-glass opacity. Minimal left pleural effusion and multiple enlarged mediastinal and hilar lymph nodes were seen. Also, minimal pericardial effusion was revealed. A core needle biopsy of the left lower lobe mass was performed. Histopathological examination revealed moderately pleomorphic epithelial cells with an oval hyperchromatic nucleus; stroma consisted of highly pleomorphic oval to spindle-shaped cells along with multinucleated malignant cells ( Figure 3). A diagnosis of PSC was made.

Epidermal growth factor receptor gene (EGFR) mutation and anaplastic lymphoma kinase (ALK) rearrangement testing was negative. Eastern Cooperative Oncology Group (ECOG) performance status of the patient was noted to be 2. The patient was not a candidate for surgical resection as it was stage IIIb (T2N3M0) disease along with multiple other comorbidities. She was started on weekly nab-paclitaxel (80 mg/m ^2^) and carboplatin (target area under the curve 2). The patient’s programmed death ligand-1 (PDL-1) expression status was not known with the initial diagnosis.

The patient’s condition quickly deteriorated within 72 hours of first cycle of chemotherapy with the development of massive pleural effusion. Therefore, the patient was started on immunotherapy with pembrolizumab 200mg intravenous, as her VENTANA PD-L1 (SP263) assay report revealed 85% tumor cells. A few days post-immunotherapy, the patient went into hypoxic respiratory failure requiring intubation. She died after two days of treatment in the intensive care unit.

## Discussion

SC of the lung is an extremely rare and poorly differentiated NSCLC, containing a sarcoma-like characteristic (malignant spindle or giant cells) or sarcomatous element (neoplastic bone, cartilage, or striated muscle) constituting of around 1% of all primary lung malignancies ( Franks & Galvin, 2010; Rajdev et al., 2018).

PSC is seen predominantly in male smokers with a higher male-to-female ratio. It is usually observed in the age group between 56 to 74 with the average age of diagnosis being 66 years ( Fishback et al., 1994; Ishida et al., 1990). The patient in our case also had a long history of smoking cigarettes and was diagnosed at the age of 68. SC of the lungs can manifest as a central or peripheral lesion, most often in the upper lobes. It grows by invading the bronchial tree, pulmonary parenchyma, and adjacent anatomical structures (mediastinum and chest wall) in the form of necrotic and hemorrhagic large masses that are round to bosselated, soft to solid, and often rubbery to stiff ( Pelosi et al., 2010). In our case, the patient had a mass in the superior segment of left lower lobe extending into the posterior basal segment with cavitation and hemoptysis as presentation.

Symptoms like cough, hemoptysis, chest pain, shortness of breath, fever, and weight loss are common. Hemoptysis occurs in about half of all cases of proximal or central tumors, whereas peripheral tumors may be asymptomatic or may present with chest pain ( Kim et al., 2002; Ouziane et al., 2014). Interstitial lung disease can co-exist, as in our case, where the patient was exposed to bio-mass fuel for a prolonged period. The tumor's morphology varies greatly on gross examination, ranging from soft to hard, or rubbery in consistency. The sliced surface ranges from whitish gray to tan-yellow, with patches of hemorrhage and necrosis ( Rajdev et al., 2018). In our case, the tumor was hard in consistency, poorly circumscribed, light brown colored tissue with patches of hemorrhage.

There are no paraneoplastic syndromes identified in PSC, despite the fact that they exist in 15–20% of small cell lung cancers and 5–8% of NSCLCs ( Jia et al., 2010). Metastasis to skin, stomach, pancreas, esophagus, jejunum, rectum, kidneys, bones and adrenal glands, brain, and mandibular gingiva have been reported with PSC ( Fernandes De Oliveira et al., 2013; Park et al., 2006). The treatment of PSC is as difficult as the diagnosis. SC, though believed to evolve from the NSCLC, behaves aggressively and presents with locally advanced or metastatic disease ( Rajdev et al., 2018). The mainstay of treatment, particularly for localized tumors, is still radical surgical resection. Radiotherapy and chemotherapy are being used as adjuvant treatment or in instances where the patient is a poor surgical candidate ( Fernandes De Oliveira et al., 2013). Similarly, the patient in our case was a poor surgical candidate due to stage IIIb (T2N3M0) disease and compromised ECOG performance status with multiple co-morbidities like interstitial lung disease, hypertension, dyslipidemia, and type 2 diabetes mellitus. She was given chemotherapy with carboplatin and nab-paclitaxel. Studies have shown that EGFR mutations can be found in 8.8% ( Li et al., 2020) and ALK rearrangement in 3.5% of SC of lung patients ( Chen et al., 2017). Our patient reported negative for both. Previous studies have reported that patients with PSC have a high rate of PD-L1 expression ( Maneenil et al., 2018). According to Velchet et al., 69.2% (9/13) of patients tested positive for PD-L1 ( Velcheti et al., 2013). Likewise, our patient was tested for PD-L1 of 85%. PSC has a poorer outcome than conventional NSCLC. SC has a 5-year survival rate of around 20%, relative to NSCLCs, which has a 5-year survival rate of 50% and the median survival time is three months ( Fernandes De Oliveira et al., 2013).

There are several limitations of our study; one of them being the lack of yearly CT scans after initial diagnosis of ILD, which hindered early diagnosis of the disease and curative treatment with local therapy like surgery. Comprehensive genomic analysis at diagnosis would have helped us to better deliver targeted therapy early on. This can be a problem in resource-limited countries like Nepal.

## Conclusion

PSC is an unusual biphasic malignant neoplasm of the lung that has a poor prognosis compared to other NSCLCs. A timely diagnosis is of vital importance to begin curative therapy, including surgery, chemotherapy, and radiotherapy. Our case highlights the aggressiveness of the disease and the importance of comprehensive investigations with a high index of suspicion. It might coexist sometimes with ILD like in our case, which should not be overlooked. Our case also highlights the timely resulting of comprehensive genomic profiling like PD-L1 which could have helped our patient. We believe that reporting this case with its overall clinical course can further add to the body of knowledge of this rare disease and aid in the development of successful treatment.

## Consent

Written informed consent for publication of their clinical details and/or clinical images was obtained from the patient’s sister.

## Data availability

All data underlying the results are available as part of the article and no additional source data are required.
