# Supplementary material for: Case Report: Pulmonary sarcomatoid carcinoma in a female patient from Nepal
Source: F1000Res. 2021 Aug 2;10:723. [Version 1] doi: 10.12688/f1000research.55187.1 (PMC8921689; doi:10.12688/f1000research.55187.1)
Supplement: Peer review history

# Peer review: Reviewer response for version 1

**DOI:** 10.5256/f1000research.58744.r118657

**Published:** 2022-03-14

**Authors:** Ramila Shilpakar

**Affiliations:** 1 Bir Hospital, National Academy of Medical Sciences, Kathmandu, Nepal

It is a nice case report by Devkota *et al. *highlighting the rare case and difficult treating scenarios in resource-limited settings. 

 The patient had hemoptysis, so why was Radiotherapy not the first choice both for disease control as well as hemoptysis? If the authors could explain this it will be helpful for further reference.

 The patient’s condition quickly deteriorated within 72 hours of the first cycle of chemotherapy with the development of massive pleural effusion: What was done to improve the dyspnea due to massive effusion, this should be written in brief to give an idea to the readers. And why was immunotherapy started rather than other supportive methods to decrease effusion related deterioration in patient condition. Please make these points clear for the readers.

Are enough details provided of any physical examination and diagnostic tests, treatment given and outcomes?

Yes

Is the case presented with sufficient detail to be useful for other practitioners?

Yes

Is sufficient discussion included of the importance of the findings and their relevance to future understanding of disease processes, diagnosis or treatment?

Yes

Is the background of the case’s history and progression described in sufficient detail?

Yes

Reviewer Expertise:

cost effective therapies

I confirm that I have read this submission and believe that I have an appropriate level of expertise to confirm that it is of an acceptable scientific standard.

# Peer review: Reviewer response for version 1

**DOI:** 10.5256/f1000research.58744.r93529

**Published:** 2021-09-15

**Authors:** Jian-Hong Fang, Yun Zhou

**Affiliations:** 1 MOE Key Laboratory of Gene Function and Regulation, School of Life Sciences, Sun Yat-sen University, Guangzhou, China; 2 State Key Laboratory of Oncology in South China, Yat-sen University Cancer Center,, Guangzhou, China

This is an interesting report on an uncommon but aggressive variant of NSCLC. The authors have in detail described the clinical course of the patients, which will benefit patients with this uncommon disease. However, more information should be provided. Specifically, after receiving nab-paclitaxel and carboplatin, the patient’s condition quickly deteriorated within 72 hours. And then, the patient was started on immunotherapy with pembrolizumab for a few days before her death. During these treatments, was any physical examination or diagnostic test conducted for this patient. If so, what were the results? This information will be helpful to reveal the cause for the rapid progress of the disease.

Are enough details provided of any physical examination and diagnostic tests, treatment given and outcomes?

Partly

Is the case presented with sufficient detail to be useful for other practitioners?

Yes

Is sufficient discussion included of the importance of the findings and their relevance to future understanding of disease processes, diagnosis or treatment?

Yes

Is the background of the case’s history and progression described in sufficient detail?

Yes

Reviewer Expertise:

The molecular mechanism underlying the tumor metastasis.

We confirm that we have read this submission and believe that we have an appropriate level of expertise to confirm that it is of an acceptable scientific standard, however we have significant reservations, as outlined above.

# Peer review: Reviewer response for version 1

**DOI:** 10.5256/f1000research.58744.r91016

**Published:** 2021-08-27

**Authors:** Alina Basnet

**Affiliations:** 1 Department of Hematology/Oncology, SUNY Upstate Medical University, Syracuse, NY, USA

This is an interesting case of an aggressive variant of NSCLC. This is unique due to the underlying history of ILD and the management in general in resource-limited countries like Nepal. 

 The clinical course has been summarised.

 If they could specify if the patient received radiation or not at any time during the course of the treatment? if not, why was the low dose chemotherapy for carboplatin and paclitaxel chosen?

 Can the authors elaborate on the staging scans that were performed? was any CT abdomen, bone scan, or PET scan done?

Are enough details provided of any physical examination and diagnostic tests, treatment given and outcomes?

Yes

Is the case presented with sufficient detail to be useful for other practitioners?

Yes

Is sufficient discussion included of the importance of the findings and their relevance to future understanding of disease processes, diagnosis or treatment?

Yes

Is the background of the case’s history and progression described in sufficient detail?

Yes

Reviewer Expertise:

lung cancer, prostate cancer, renal cell carcinoma, bladder cancer

I confirm that I have read this submission and believe that I have an appropriate level of expertise to confirm that it is of an acceptable scientific standard.

# Response

**Published:** 2021-09-02

**Authors:** Anirudra Devkota

Radiotherapy was planned but couldn't be performed as the patient rapidly deteriorated. Low dose chemotherapy was preferred because our patient was elderly, and also the general condition was poor. We couldn't perform the Bone scan and PET Scan as it was not available in our hospital. Diagnostic centers were also closed due to COVID lockdown. CT chest together with upper abdomen was done, but CT abdomen separately was not performed.
